# Characteristics of CpG Islands and their quasispecies of full-length hepatitis B virus genomes from patients at different phases of infection

**DOI:** 10.1186/s40064-016-3192-3

**Published:** 2016-09-21

**Authors:** Yuan Xue, Ming-Jie Wang, Su-Yuan Huang, Zhi-Tao Yang, De-Min Yu, Yue Han, Ming-Yu Zhu, Dao Huang, Dong-Hua Zhang, Qi-Ming Gong, Xin-Xin Zhang

**Affiliations:** 1 Clinical Virology Research Laboratory, Ruijin Hospital, Shanghai Jiaotong University, School of Medicine, No. 197, Ruijin Er Road, Shanghai, 200025 China; 2 Department of Infectious Diseases, Institute of Infectious and Respiratory Diseases, Ruijin Hospital, Shanghai Jiaotong University, School of Medicine, Shanghai, China; 3 Translational Medicine Research Center, Ruijin Hospital North, Shanghai Jiaotong University, School of Medicine, Shanghai, China; 4 Pôle Sino-Français de Recherches en Science du Vivant et Génomique, Rui Jin Hospital, Shanghai Jiaotong University, School of Medicine, Shanghai, China

**Keywords:** CpG islands, Genotype, Genetic heterogeneity, Hepatitis B virus

## Abstract

**Background:**

CpG islands in hepatitis B virus (HBV) genome are potential targets for methylation mediated gene silencing, and may be involved in the pathogenesis of HBV infection. To date, their characteristics in HBV quasispecies (QS) remain largely unknown. The purpose of this study was to investigate the characteristics of CpG islands in HBV QS.

**Methods:**

Forty patients diagnosed as acute hepatitis B (AHB, n = 10), immune-tolerant HBV carriers (IT, n = 9), chronic hepatitis B (CHB, n = 11), or acute on chronic liver failure (ACLF, n = 10), were enrolled in this case–control study. A total of 599 clones were isolated, and full-length HBV genomes were sequenced.

**Results:**

CpG island II (CGII) in AHB group was shorter in length and its QS heterogeneity was lower than that in the chronic infection group. Among the chronic infection subgroups, CGII and CpG island III (CGIII) in IT group were longer and their heterogeneity was lower compared to CHB and ACLF groups. Length of CGII correlated with HBV DNA levels positively while the complexity and diversity of CGII correlated with HBV DNA levels negatively. Moreover, CGII and CGIII were shorter in genotype B than those in genotype C, while QS complexity and diversity of either CGII or CGIII had no significant difference between genotype B and C.

**Conclusions:**

Overall, our results suggest that the distribution, length and QS heterogeneity of CpG islands in full-length HBV genome differ across clinical phases of infection, of which the mechanism warrants further study.

**Electronic supplementary material:**

The online version of this article (doi:10.1186/s40064-016-3192-3) contains supplementary material, which is available to authorized users.

## Background

Hepatitis B virus (HBV) infection is a challenging health problem and a leading cause of liver diseases in Asia–Pacific region. It is estimated that approximately 240 million people are chronically infected worldwide, and are at risk of developing end-stage liver diseases (Ott et al. 2012). Although the molecular mechanisms determining persistent infection are not fully elucidated, it is generally accepted that both the virus genome and host immune system contribute to the outcomes of infection.

CpG islands which are CpG-rich regions in HBV genome, are potential targets for methylation mediated gene silencing and are related with virus replication (Vivekanandan et al. 2010). There are three conventional CpG islands termed CpG island I (CGI), CpG island II (CGII) and CpG island III (CGIII) (Zhang et al. 2013). CGI (nt67 ~ nt212) is located in the start site of the S region, and CGII (nt1170 ~ nt1671) overlaps the enhancer I and the promoter of X region, while CGIII (nt2280 ~ nt2455) covers the partial C gene and encompasses the start site of the P region. Different distribution of CpG islands which can affect their methylation status and HBV gene expression, might further lead to different clinical outcomes of HBV infection. Previous studies have shown that distribution of CpG islands differed across HBV genotypes (Hou et al. 2015; Zhang et al. 2013; Zhong et al. 2015). However, all of the nucleotide sequences mentioned in those studies were searched from Genbank at the National Center for Biotechnology Information. To date, there is no datum from real-life study to explore the characteristics of CpG islands in HBV genome from different phases of infection.

It is worth noting that HBV exists as a spectrum of strains. Due to a high replication rate and lack of proofreading activity during reverse transcription, HBV exists as quasispecies (QS), including variants which are genetically distinct, but closely related (Ngui and Teo 1997). Because of the different adaptability, QS are related to the outcome of HBV infection (Cao et al. 2014; Yang et al. 2015) and antiviral response (Liu et al. 2011; Chen et al. 2009; Cheng et al. 2013; Peveling-Oberhag et al. 2013; Tong et al. 2013). Collectively, the characteristics of CpG islands in HBV QS isolated from real-life patients remain largely unknown.

In the present study, 599 clones from forty patients were isolated, and full-length HBV genomes were sequenced. Characteristics of CpG islands, including the distribution, length and heterogeneity in HBV QS, were investigated for better understanding the role of HBV in pathogenesis.

## Materials and methods

### Patients

Forty treatment-naïve patients with HBV infection from Shanghai Ruijin Hospital were retrospectively enrolled in our present study. These patients were enrolled from September, 2009 to January, 2014. Among 40 patients, 10 patients were diagnosed as acute hepatitis B (AHB), 9 patients in high replicative, low inflammatory phase (previously termed “immune-tolerant HBV carriers”, IT) (Gish et al. 2015), 11 patients with chronic hepatitis B (CHB), and the other 10 with acute on chronic liver failure (ACLF). AHB is defined as a transient presence of HBsAg within 6 months without previous history of chronic hepatitis B. Diagnosis of IT, CHB and ACLF was according to criteria recommended by the Asian Pacific Association for the Study of the Liver (APASL) (Sarin et al. 2009; Liaw et al. 2012). IT is defined as HBeAg positive with high levels of HBV DNA (>2,000,000 IU/ml) but have normal serum alanine aminotransferase (ALT) (Liaw et al. 2012), while inclusion criteria of CHB group are HBsAg positive for more than 6 months and HBV DNA level >20,000 IU/ml with serum ALT level more than 2 ULN, in the present study (Yang et al. 2015). ACLF was defined as an acute hepatic insult manifesting as jaundice, coagulopathy, complicated within 4 weeks by ascites and/or encephalopathy in patients with previously diagnosed chronic HBV infection (Sarin et al. 2009). Sera of AHB and ACLF patients were collected during the first week of clinical onset. Patients were excluded if they were diagnosed as autoimmune liver disease, alcoholic liver disease, or co-infection, such as human immunodeficiency virus, hepatitis C virus, hepatitis D virus, Epstein-Barr virus, cytomegalovirus. Sera were collected from clinical detecting laboratory after completing clinical tests. The remaining sera were collected and froze in −80 °C refrigerator.

### Compliance with ethical standards

The study was a non-invasive and non-interventional retrospective study. The study was fully anonymous, thus it cannot do harm to the patients. The study was approved by the Ethics Committee of Ruijin Hospital in accordance with the Declaration of Helsinki.

### Liver biochemistry, HBV serological markers and HBV DNA tests

Liver biochemistry indexes were tested using an automated chemistry analysis system (Beckman Coulter, Fullerton, CA, USA). HBV serological markers including HBsAg, anti-HBs, HBeAg, anti-HBe and anti-HBc were determined by chemiluminescent microparticle enzyme immunoassay using the Abbot Architect immunoassay system (Abbort Laboratories, Abbott Park, IL, USA). The HBV DNA levels were measured by PCR using the Cobas Amplicor HBV Monitor Test (Roche Diagnostics, Mannheim, Germany) with a low detection limit of 60 IU/ml.

### Molecular cloning and sequencing

As described in our previous study (Yang et al. 2015), HBV DNA was extracted from 200 μl serum at baseline (before treatment) using the QIAamp blood mini-kit (Qiagen, Hilden, Germany) according to the manufacturer’s instructions. The full-length HBV genomes were amplified by PCR as Gunther described (Gunther et al. 1995). The primers were as follows: 5′-TTT TTC ACC TCT GCC TAA TCA-3′ (forward, nt 1821–1841) and 5′-AAA AAG TTG CAT GGT GCT GG-3′ (reverse, nt 1825–1806). PCR products of about 3200 bp were purified using the QIAquick Gel Extraction Kit (QIAgen Hilden, Germany), and cloned into the pGEM-T vector after the addition of adenylate-tail (Promega, Madison, WI, USA), and then transformed into TOP10 *Escherichia coli* competent cells (Invitrogen, Carlsbad, CA) growing on ampicillin plates. An average of 15 (range from 14 to 17) positive clones per sample were sequenced using an ABI 3730 automated sequencer (Applied Biosystems, Foster City, CA, USA). A total of 599 clones from 40 patients were sequenced.

### QS heterogeneity analysis

All sequences were aligned using CLUSTAL X (version 2.0 (Thompson et al. 1997); RDP3 software was used to detect recombinant sequences which were excluded (Martin and Rybicki 2000; Liu et al. 2011). Sequence segments were assembled to full-length HBV using Codon Code Aligner 3.7.1 software package (Codon Code Corporation, Dedham, MA). Genotypes of each sequence were determined using the HBV STAR program online(Myers et al. 2006). Viral QS heterogeneity was evaluated with complexity and diversity. QS complexity refers to the distribution of different mutant genomes in a population, and it is calculated using normalized Shannon entropy (Sn) formula as previously described (Liu et al. 2011; Domingo et al. 2006; Chen et al. 2009). QS diversity was defined as the relatedness of individuals within the population, and it was evaluated by the mean genetic distance (d, 10^−3^ subsituation/site) using MEGA5.0 software (Tamura. 1992; Tamura et al. 2007).

### CpG islands analysis

CpG islands were analyzed using the MethPrimer (http://www.urogene.org/cgi-bin/methprimer/methprimer.cgi) and the CpG Plot (http://www.ebi.ac.uk/Tools/emboss/cpgplot). The CpG islands were defined according to three criteria (Fazzari and Greally 2004; Zhang et al. 2013): (1) a GC content of ≥50 %, (2) an observed-to-expected CpG dinucleotide ratio ≥0.60, and (3) a sequence longer than 100 bp. The distribution, length, and QS heterogeneity of CpG islands within each clone were obtained.

### Statistical analysis

Length and QS complexity of CpG islands were expressed as mean ± SE, while the mean genetic distance was expressed as median with range. Results of continuous variables were compared between acute and chronic infection groups by unpaired t test or the Mann–Whitney test, and variables were compared between the chronic subgroups by one-way ANOVA analysis of variance or Kruskal–Wallis test as needed. Proportion of diseases was compared using Chi Square tests. Correlations were analyzed using Pearson correlation analysis. All analyses were performed using SPSS19.0 software (Chicago, IL, USA). Differences were considered significant at a *P* value <0.05.

## Results

### Clinical and laboratory data of patients

The demographics, clinical and laboratory data are shown in Table 1. All AHB patients had spontaneous HBsAg/anti-HBs seroconversion, and five in ten ACLF patients died within 3 months after the clinical onset of infection. The proportions of patients harboring HBV genotype B and C did not differ significantly among the four groups (*P* > 0.05).

**Table 1:** Demographic and clinical features of patients (mean ± SE)

	AHB (n = 10)	IT (n = 9)	CHB (n = 11)	ACLF (n = 10)	*P* value
Sex (M/F)	9/1	5/4	6/5	10/0	0.032
Age (year)	40.70 ± 2.62	28.56 ± 1.84^a,b^	35.55 ± 3.89	43.80 ± 2.82	0.008
ALT (U/L)	1608.5 ± 199.29	29.67 ± 3.76^a,b^	263.45 ± 131.04^c,d^	1171.57 ± 184.49	0.001
TBIL (μmol/L)	327.62 ± 130.01	16.01 ± 1.40^a,b^	32.95 ± 9.36^c,d^	301.79 ± 29.83	0.002
PTA (%)	77.89 ± 5.45	103.33 ± 3.33	98.79 ± 1.21	30.58 ± 2.65^b,d,e^	0.000
HBeAg (±)	10/0	9/0	10/1	2/8^b,d,e^	0.000
HBV DNA (log_10_IU/ml)	6.55 ± 0.45	8.02 ± 0.19^a,b^	7.09 ± 0.38	6.34 ± 0.26	0.011
Genotype (B/C)	4/6	3/6	6/5	5/5	0.777

The proportions of patients harboring HBV genotype B and C did not differ significantly among the four groups (*P* > 0.05)

*AHB* acute hepatitis B, *IT* immune-tolerance, *CHB* chronic hepatitis B, *ACLF* acute on chronic liver failure, *ALT* alanine aminotransferase, *TBIL* total bilirubin, *PTA* prothrombin time activity

^a^ *P* < 0.01 IT versus AHB, ^b^ *P* < 0.01 IT versus ACLF, ^c^ *P* < 0.01 CHB versus AHB, ^d^ *P* < 0.01 CHB versus ACLF, ^e^ *P* < 0.01 AHB versus ACLF

### Distribution of CpG islands in patients with different phases of infection

**Fig. 1:**
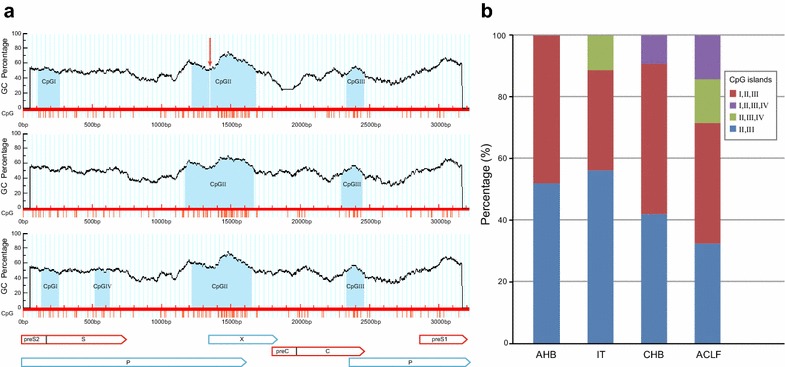
Distribution of CpG islands in HBV genome. **a** The *vertical axes* indicate the GC percentage, and the *horizontal axes* represent the HBV nucleotide sequence. The *blue areas* refer to the CpG islands, while the *vertical red lines* under the *horizontal axes* represent CpG dinucleotides. The *horizontal arrows* represent the open reading frames of preS1/preS2/S, X, preC/C and p genes, while the *vertical arrow* indicates the split CGII. CGI, CGII and CGIII coexist in the first graph, while CGI is absent in the second graph, and CGIV is present in the third graph. **b** The frequency of strains containing CGIV in AHB, IT, CHB and ACLF group was compared by Chi Square test. CGIV was much more common in ACLF group compared to the other three groups (χ^2^ = 59.76*, P* < 0.01)

Experiments of molecular cloning and sequencing were performed in our previous study (Yang et al. 2015), and the characteristics of CpG islands in full-length HBV genomes of 599 clones were analyzed and new data were generated in the present study (Genbank submission numbers: KU963799–KU964397). The distribution of CpG islands in HBV-related liver diseases is shown in Fig. 1. All clones contained CGII and CGIII. Besides the three conventional CpG islands, CpG island IV (CGIV, nt332 ~ nt632) was identified. The frequency of strains containing CGIV in AHB, IT, CHB and ACLF group was 0 % (0/146), 11.19 % (15/134), 9.09 % (15/165) and 28.57 % (44/154), respectively. Strikingly, CGIV was much more common in ACLF group, compared to the other three groups (χ^2^ = 59.76, *P* < 0.01) (Fig. 1b).

### QS characteristics of CpG islands in patients with acute and chronic infection

**Fig. 2:**
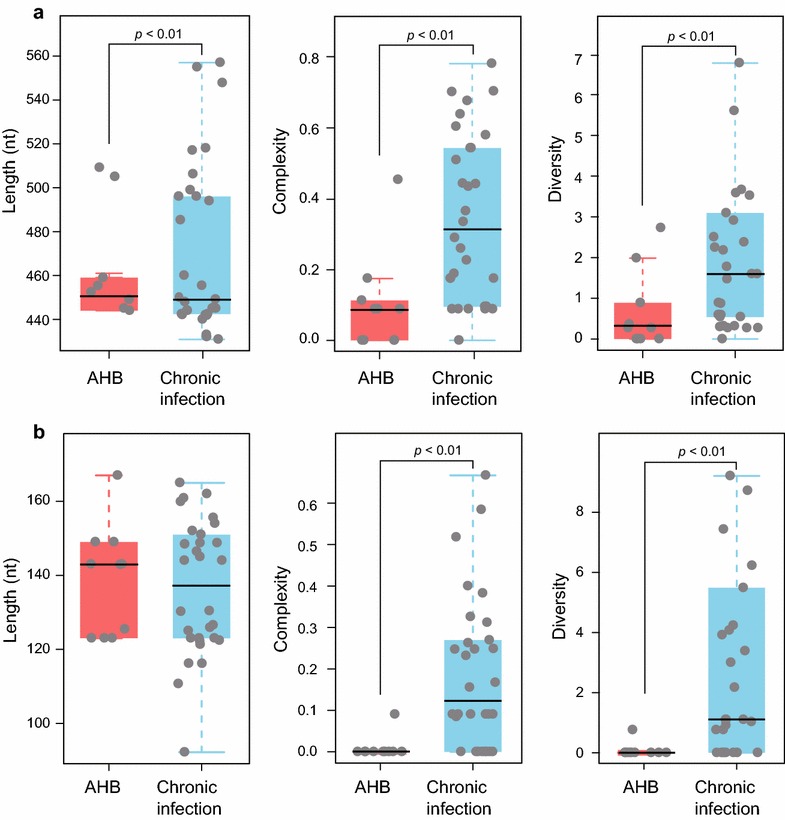
QS characteristics of CpG islands in patients with acute and chronic infection. **a** CGII in AHB group was statistically shorter in length, and its QS complexity and diversity were lower than those in the chronic infection group. **b** QS complexity and diversity of CGIII were lower in AHB group than those in the chronic infection group

In an attempt to investigate the characteristics of CpG islands among different phases of infection, lengths and QS heterogeneity of CpG islands were compared (data shown in the Additional file 1: Table S1). As shown in Fig. 2a, length of CGII in AHB group was statistically shorter than that in the chronic infection group (*P* < 0.01). QS complexity and diversity of CGII in AHB group were lower than those in the chronic infection group (*P* < 0.01).

As for CGIII, there was no significant difference in length between acute and chronic infection groups. Similar to CGII, complexity and diversity of CGIII in AHB group were lower than those in the chronic infection group (*P* < 0.01) (Fig. 2b).

### QS characteristics of CpG islands in patients with chronic infection

**Fig. 3:**
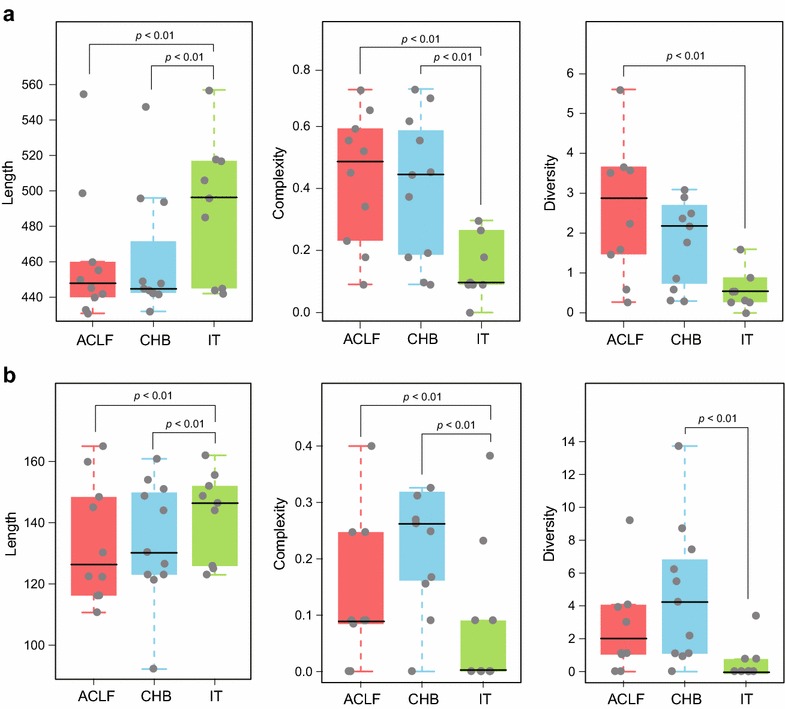
QS characteristics of CpG islands in patients with chronic infection. Length of CGII (**a**) and CGIII (**b**) were longer and QS complexity was lower in IT subgroup compared to the CHB and ACLF subgroups

Comparison of QS characteristics among chronic infection subgroups is shown in Fig. 3. Among the subgroups, IT patients had longer CGII than CHB and ACLF patients. Compared to CHB and ACLF subgroups, QS complexity of CGII in IT subgroup was lower. As for QS diversity, it was lower in IT subgroup than that in ACLF subgroup.

Similar to CGII, CGIII in IT subgroup was the longest, and its QS heterogeneity was the lowest, while there was no significant difference between CHB and ACLF subgroups.

### HBV DNA levels correlate with the length and QS heterogeneity of CpG island II

**Fig. 4:**
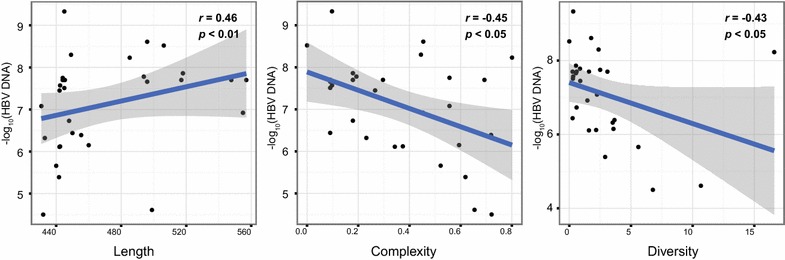
HBVDNA levels correlate with the length and QS heterogeneity of CpG island II. HBV DNA levels correlated positively with the length of CGII, and negatively with QS complexity and diversity of CGII

Pearson correlation analysis revealed a positive correlation between HBV DNA levels and the length of CGII (r = 4.66, *P* < 0.01), while HBV DNA levels correlated negatively with QS complexity (r = −4.54, *P* < 0.05) and diversity (r = −4.31, *P* < 0.05) of CGII (Fig. 4).

### QS characteristics of CpG islands in HBV genotype B and C

Consistent with the previous studies (Zhang et al. 2013; Zhong et al. 2015; Hou et al. 2015), CGI and split CGII were much more common in HBV genotype B compared to genotype C. Nevertheless, 30 clones in genotype C from two patients (one CHB patient and one ACLF patient) contained CGI, while among the 16 clones in genotype B from a CHB patient, only three clones contained CGI.

**Fig. 5:**
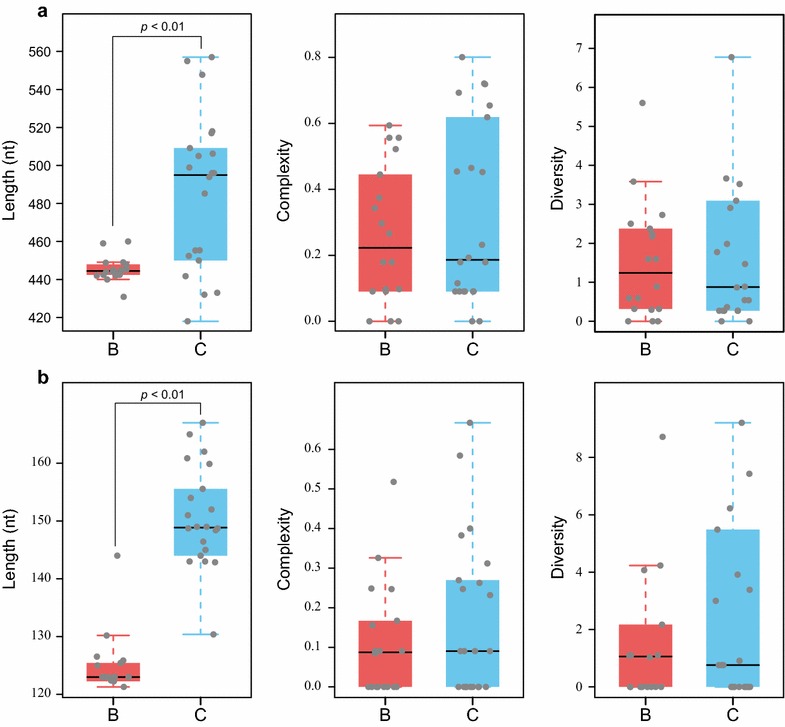
QS characteristics of CpG islands in HBV *genotype B* and *C*. CGII (**a**) and CGIII (**b**) in *genotype B* were statistically shorter than those in *genotype C*, while QS complexity and diversity of either CGII or CGIII had no significant difference between *genotype B* and *C*

As shown in Fig. 5, both CGII and CGIII in HBV genotype B were shorter than those in genotype C (*P* = 0.000 and 0.000, respectively). QS complexity and diversity of either CGII or CGIII had no significant difference between genotype B and C (*P* > 0.05). Pearson correlation analysis showed that QS complexity did not correlate with the length of CpG islands (*P* > 0.05).

## Discussion

In the present study, characteristics of CpG islands in HBV QS were investigated in real-life study for the first time. The results indicated that CGII in AHB group was shorter in length and its QS heterogeneity was lower than that in the chronic infection group. Among the chronic infection subgroups, CGII and CGIII in IT subgroup were longer and their heterogeneity was lower compared to CHB and ACLF subgroups. Moreover, HBV DNA levels correlated positively with the length of CGII, and negatively with the heterogeneity of CGII.

The full-length HBV sequence is needed for CpG islands analysis. The sequence obtained by splicing various PCR-amplified fragments, maybe is not a real existing full-length sequence because of the HBV QS. The classic method reported by Gunther et al. (1995) is still the gold standard for complete HBV genome study. In accordance with Gunther, all HBV strains in our present study were obtained by full-length cloning/sequencing technique instead of splicing PCR-amplified fragments.

For the first time, we compared the QS characteristics of CpG islands from patients with different outcomes of infection. It is generally considered that patients with acute self-limited infection have effective immune response to eliminate virus, while immune-tolerant patients have high levels of HBV replication and lack inflammation in the liver. The mechanisms of different outcomes caused by HBV infection are considered to be multi-factorial, including the immune suppression and virus factors (Cao et al. 2014). In the present study, QS complexity of CGII in CHB and ACLF groups was higher than that in IT groups. HBV QS evolution from immune-tolerance to immune-active phases may result from the immune selection.

CGII which overlaps the enhancer I and the X gene promoter, is an important region for the regulation of HBV transcription and replication (Guo et al. 2011). It has been reported that HBV core protein (HBc) binds to HBV cccDNA preferentially at the CGII region (Guo et al. 2011). The frequency of HBc binding to CGII is positively correlated with the ratio of relaxed circular DNA to cccDNA and the levels of serum HBV DNA (Guo et al. 2011). Another study reported that promoters with long CpG islands encoded more RNA polymerase II binding sites than that with short ones (Elango and Yi 2011). CGII in AHB group is shorter than that in the chronically infected group, and is longer in IT subgroup compared to CHB and ACLF subgroups. An interesting finding of our study is that HBV DNA levels correlate positively with the length of CGII, and negatively with the complexity of CGII. CGII, whose length is related to viral replication, may play a role in the clinical outcomes of HBV infection. Our study can partially elucidate mechanisms underlying the different outcomes of HBV infection.

Genotype B and C, which are two major prevalent HBV genotypes in China (Zeng et al. 2005; Chu and Liaw 2005), have a divergence of more than 8 % in the complete genome nucleotide sequence, and different clinical characteristics. In general, infection with HBV genotype B is apt to have HBV e antigen seroconversion, while infection with genotype C is associated with higher risk of developing cirrhosis and HCC compared to genotype B (Chu and Liaw 2005; Kong et al. 2014; Tseng and Kao 2008; Malmstrom et al. 2012). To date, the mechanism for the differences remains unclear. Our present study showed that CGI was more common and lengths of CGII were shorter in genotype B compared to genotype C. Since CGI overlaps the forepart of S gene, the different distribution of CGI between genotype B and C may affect the methylation of CpG islands, and influence the regulation of HBV gene expression, especially the S gene. Although the function of CGI remains unclear, its absence might induce less methylation of the first CpG-rich region in HBV genotype C (Zhang et al. 2013), and likely to be associated with the progressive liver diseases caused by HBV genotype C. To note, as shown in the present study, 30 clones in genotype C from two patients (one CHB patient and one ACLF patient) contained CGI, while among the 16 clones in genotype B from a CHB patient, only three clones contained CGI. Different from the previous studies (Hou et al. 2015; Zhang et al. 2013; Zhong et al. 2015), data from HBV quasispecies emphasize that, distribution of CpG islands does not abide by genotypes strictly.

Moreover, little is known about whether HBV genotypes affect the QS heterogeneity. Data from this real-life study showed that QS complexity and diversity of either CGII or CGIII had no significant difference between genotype B and C. Therefore, QS heterogeneity may not account for the different clinical outcomes between genotype B and C.

## Conclusions

To our knowledge, this is the first real-life study that explored the QS characteristics of CpG islands in full-length HBV genome. Obtained data demonstrate that lengths and QS heterogeneity of CpG islands differ across clinical phases of infection. These data may partially explain the different clinical characteristics among clinical phases of infection, of which the mechanism warrants further study.

## Additional file

10.1186/s40064-016-3192-3 Lengths and quasispecies heterogeneity of CpG islands in patients (mean ± SD).

## Abbreviations

HBV: hepatitis B virus
QS: quasispecies
AHB: acute hepatitis B
IT: immune-tolerant HBV carrier
CHB: chronic hepatitis B
ACLF: acute on chronic liver failure
CGI: CpG island I
CGII: CpG island II
CGIII: CpG island III
CGIV: CpG island IV
ALT: serum alanine aminotransferase
Sn: Shannon entropy
