# Supplementary material for: Characteristics of CpG Islands and their quasispecies of full-length hepatitis B virus genomes from patients at different phases of infection
Source: Springerplus. 2016 Sep 21;5(1):1630. doi: 10.1186/s40064-016-3192-3 (PMC5031574; doi:10.1186/s40064-016-3192-3)
Supplement: Supplementary file 1 — 10.1186/s40064-016-3192-3 Lengths and quasispecies heterogeneity of CpG islands in patients (mean ± SD). [file 40064_2016_3192_MOESM1_ESM.docx]

**Table S1. Lengths and quasispecies heterogeneity of CpG islands in patients (mean±SD).**

|  | AHB | Chronic infection | | | |
| --- | --- | --- | --- | --- | --- |
|  | (n=10) | Total | IT(n=9) | CHB(n=11) | ACLF(n=10) |
| **CpG island II** |  |  |  |  |  |
| Numbers (clones) | 146 | 453 | 134 | 165 | 154 |
| Lengths (bp) | 445.24±50.09^a^ | 470.39±40.38 | 489.69±41.89^b,c^ | 462.36±34.78 | 462.21±39.14 |
| Complexity | 0.1031±0.1413^a^ | 0.3558±0.2411 | 0.2124±0.2398^b,c^ | 0.4019±0.2335 | 0.4342±0.2135 |
| d(10^-3^ substitutions/site) | 0.2699(0～2.7285)^a^ | 1.5952(0～16.6141) | 0.5397(0～16.6141)^c^ | 2.1781(0.2964～6.7762) | 2.8833(0.2700～10.6687) |
| **CpG island III** |  |  |  |  |  |
| Numbers (clones) | 146 | 453 | 134 | 165 | 154 |
| Lengths (bp) | 139.07±14.39 | 136.36±18.61 | 142.52±14.02^b,c^ | 133.96±20.05 | 133.57±19.35 |
| Complexity | 0.0090±0.0286^a^ | 0.1882±0.1847 | 0.0883±0.1352^b,c^ | 0.2802±0.1974 | 0.1768±0.1730 |
| d(10^-3^ substitutions/site) | 0 (0～0.7597)^a^ | 1.1019(0～26.1658) | 0 (0～14.4352)^b^ | 4.2328(0～13.7355) | 2.0510(0～26.1658) |

AHB, acute hepatitis B; IT, immune-tolerant HBV carrier; CHB, chronic hepatitis B; ACLF, acute on chronic liver failure; d, the mean genetic distance at nucleotide level. Unpaired *t* test was performed when analyzing the length and complexity of CpG islands, and Mann-Whitney test was performed when analyzing the mean genetic distance between acute and chronic infection groups. One-way ANOVA of variance was performed when analyzing the length and complexity of CpG islands, and Kruskal-Wallis test was performed when analyzing the mean genetic distance between the three chronic infection subgroups. ^a^*P*＜0.01, AHB *vs.* Chronic infection; ^b^*P*＜0.01, IT *vs.* CHB ; ^c^*P*＜0.01, IT *vs.* ACLF.
